# Brain Stimulation in Alzheimer's Disease

**DOI:** 10.3389/fpsyt.2018.00201

**Published:** 2018-05-22

**Authors:** Chun-Hung Chang, Hsien-Yuan Lane, Chieh-Hsin Lin

**Affiliations:** ^1^Institute of Clinical Medical Science, China Medical University, Taichung, Taiwan; ^2^Department of Psychiatry & Brain Disease Research Center, China Medical University Hospital, Taichung, Taiwan; ^3^Graduate Institute of Biomedical Sciences, China Medical University, Taichung, Taiwan; ^4^Department of Psychology, College of Medical and Health Sciences, Asia University, Taichung, Taiwan; ^5^Kaohsiung Chang Gung Memorial Hospital, Chang Gung University College of Medicine, Kaohsiung, Taiwan

**Keywords:** brain stimulation, Alzheimer's disease (AD), transcranial magnetic stimulation (TMS), transcranial direct current stimulation (tDCS), transcranial alternating current stimulation (tACS), electroconvulsive treatment (ECT), magnetic seizure therapy (MST), cranial electrostimulation (CES)

## Abstract

Brain stimulation techniques can modulate cognitive functions in many neuropsychiatric diseases. Pilot studies have shown promising effects of brain stimulations on Alzheimer's disease (AD). Brain stimulations can be categorized into non-invasive brain stimulation (NIBS) and invasive brain stimulation (IBS). IBS includes deep brain stimulation (DBS), and invasive vagus nerve stimulation (VNS), whereas NIBS includes transcranial magnetic stimulation (TMS), transcranial direct current stimulation (tDCS), transcranial alternating current stimulation (tACS), electroconvulsive treatment (ECT), magnetic seizure therapy (MST), cranial electrostimulation (CES), and non-invasive VNS. We reviewed the cutting-edge research on these brain stimulation techniques and discussed their therapeutic effects on AD. Both IBS and NIBS may have potential to be developed as novel treatments for AD; however, mixed findings may result from different study designs, patients selection, population, or samples sizes. Therefore, the efficacy of NIBS and IBS in AD remains uncertain, and needs to be further investigated. Moreover, more standardized study designs with larger sample sizes and longitudinal follow-up are warranted for establishing a structural guide for future studies and clinical application.

## Introduction

Alzheimer's disease (AD) is a progressive neurodegenerative disorder and accounts for most of dementia in the elderly (1, 2). The prevalence of dementia due to AD in adults aged more than 60 years was 4.02% (3). 35.6 million adults were victims of dementia in the world in 2010, and the number is estimated to be 65.7 million in 2030 (4). AD is costly, with worldwide spending estimated to be US $422 billion in 2009 (5). Currently, cholinesterase inhibitors and N-methyl-D-aspartate receptor partial antagonist, memantine, are the main pharmacologic treatments for patients with AD. However, these treatments are accompanied by adverse effects and the response is limited (6). Therefore, alternative treatments require urgent development.

The use of brain stimulation has recently garnered considerable clinical and academic interest. In this review, we explore invasive brain stimulation (IBS), non-invasive brain stimulation (NIBS), and their potential applications in the AD field. IBS includes deep brain stimulation (DBS) and invasive VNS. NIBS includes transcranial magnetic stimulation (TMS), transcranial direct current stimulation (tDCS), transcranial alternating current stimulation (tACS), electroconvulsive treatment (ECT), magnetic seizure therapy (MST), cranial electrostimulation (CES), and non-invasive vagus nerve stimulation (VNS).

## Invasive stimulation methods

### Deep brain stimulation

A deep brain stimulation (DBS) system includes electrode leads, wires, and a pulse generator. Neurosurgeons implant electrode leads in the brain and pulse generator below the collar bone. Both are connected by wires which are tunneled underneath the skin. To date, DBS is the Food and Drug Administration (FDA)-approved for the management of Parkinson's disease, and essential tremor. In addition, this device has also been approved for refractory obsessive-compulsive disorder and dystonia symptoms as a humanitarian device exemption (7–10).

The first DBS trial for AD was performed in 1984, and the targeted brain region was the nucleus basalis of Meynert (NBM). Turnbull and colleagues found no improvement in memory or cognition, but the researchers noted preserved cortical glucose metabolic activity in the left parietal and left temporal lobes as well as the partial arrest of deterioration in the left frontal area (11). However, no subsequent trials of DBS for AD were performed for 26 years. When using DBS of the fornix to treat obesity in 2008, Hamani et al. discovered “deja vu-like” sensations during surgery. The researchers also found improvements in spatial associative learning and verbal learning after 3 weeks of DBS treatment (12). Therefore, in 2010, a Phase I trial was implemented to investigate DBS treatment of the fornix/hypothalamus in six adults with early AD. Two patients experienced autobiographical experiential phenomena during surgery. Moreover, after 12-month DBS treatment, the patients exhibited improved memory, reduced cognitive decline, reversed glucose metabolism (13), and increased hippocampal volume (14).

Because the Phase I trial demonstrated the promising effects of DBS of the fornix, the same group enrolled 42 participants with mild, probable AD for Phase II study (15). In this randomized double-blind trial, 21 participants in “off stimulation group” did not receive stimulation, whereas 21 participants in “on stimulation group” underwent continuous DBS stimulation for 12 months. Subsequently, all participants receive stimulation for 12 months. However, the first year of this trial revealed no significant differences of cognitive scores between these two groups. Moreover, this trial revealed that the cognitive function of patients aged < 65 years significantly worsened after 1 year of DBS, whereas patients aged ≥65 exhibited a slight improvement in cognitive function. In terms of safety, the authors observed four acute serious safety events and three long-term serious events, and suggested DBS was well-tolerated (15).

In addition to the Phase I (13) and Phase II trials (15) of fornix DBS in North America, DBS for AD studies have also been conducted in Europe. In France, Fontaine et al. reported that after 12 months of fornix DBS, a patient with mild cognitive decline showed stabilized cognitive performance and increased mesial temporal lobe metabolism (16). In Germany, Kuhn and colleagues delivered bilateral DBS over the NBM of 6 participants with mild to moderate AD (17). The authors used the Alzheimer's Disease Assessment Scale-Cognitive subscale (ADAS-Cog) to evaluate the patients' cognition. Four of the six patients were considered responders 12 months after surgery. Moreover, several participants exhibited increased temporal and amygdalohippocampal glucose metabolism after stimulation for almost 1 year. Based on these promising findings, Kuhn et al. performed continuous DBS of the NBM in two patients of an average age younger than that of the patients in the aforementioned Phase I trial, and who both had lower baseline ADAS-Cog scores (18). One patient's cognition had worsened after 26 months according to ADAS-Cog and Mini–Mental State Examination (MMSE) scores, whereas the other patient had a stable ADAS-Cog score and improved MMSE score after 28 months. Hardenacke et al. (19) evaluated the findings of the Phase I trial (17) and 2 new patients and suggested that performing DBS of the NBM at a younger age and earlier disease stage may favorably influence cognitive function and disease progression.

### Invasive vagus nerve stimulation

Vagus nerve stimulation (VNS) modulates brain network activity by stimulating tenth cranial nerve. The stimulation of tenth cranial nerve (vagus nerve) can be performed using two methods: direct invasive stimulation and indirect transcutaneous non-invasive stimulation. The invasive VNS (iVNS) system includes a pulse generator and electrodes. Surgeons attach electrodes to the left-side vagus nerve and connect them to the pulse generator which is implanted in the left thoracic region. The pulse generator delivers programmable electrical stimulation to the vagus nerve (20, 21).

Two studies have investigated the relationship between iVNS and AD (21, 22). In Sweden, researchers enrolled 10 patients with AD (22). Each patient received surgery to implant a pulse generator, which deliver programmable signals to the left-side tenth cranial nerve. The initial settings were frequency 20 Hz, pulse width 500 μs, and current 0.25 mA. The stimulation was on for 30 s followed by a pause for 5 min. After 3 months of treatment, they found the response rates were 70% (seven of 10 patients) on the ADAS-Cog and 90% (nine of 10 patients) on the MMSE. The response rates remained similar after 6 months of the iVNS treatment. Adverse effects of iVNS were transient and mild.

Therefore, based on these promising findings, the same research team recruited another seven patients with AD. The researchers followed up these total 17 patients for at least 1 year (21). They found that 1 year after iVNS treatment, the score of the ADAS-Cog in seven patients did not decline or even increase, while the score of the MMSE in 12 patients did not decline or even improve compared with baseline. These two small trials revealed that invasive VNS was well-tolerated and improved specific cognitive functions in MMSE and ADAS-Cog after 1 year and 3 months of treatment, respectively.

## Non-invasive stimulation methods

### Transcranial magnetic stimulation

In 1985, Barker and colleagues first reported transcranial magnetic stimulation (TMS) on motor cortex (23). TMS delivers a rapidly changing current through a coiled wire encased in plastic above the scalp. Based on Faraday's law of electromagnetic induction, this results in a magnetic field across the skull, and subsequently generate an electric current in the targeted brain regions (24, 25). The stimulation intensity determines the dosage according to the individual's motor-evoked potential threshold, and modulates the cortical neurons (26). Repetitive TMS (rTMS) delivers trains of several pulses at the same intensity over a period of time. rTMS protocols comprises high frequency (≥ 5 Hz) and low-frequency (≤ 1 Hz) as well as various types of stimulation bursts such as theta-burst stimulation (TBS) (25).Generally, higher frequencies (e.g., 20 Hz) may increase cortical excitability, whereas lower frequencies (approximately 1 Hz) may inhibit cortical excitability (24–26). However, low-frequency TMS may not always result in inhibition (27). Moreover, in the motor cortex, continuous TBS causes inhibitory aftereffects, whereas intermittent TBS causes excitatory aftereffects (28). Different stimulation protocols produce different aftereffects of different durations. For example, TBS protocols yield the longest aftereffects of up to 8 h, whereas long or multiple rTMS trains yield aftereffects of less than 1 h (29, 30).

Several trials and reviews have suggested that rTMS may be beneficial for various cognitive functions in patients with AD (31–41). In Italy, Cotelli and colleagues recruited 15 patients with AD and reported that rTMS administered to the bilateral dorsolateral prefrontal cortexes (DLPFCs) enhanced accuracy in action naming (31). Based on this promising finding, this research team launched another trial of 24 adults with AD of varying severity (a mild AD group and moderate-to-severe AD group) (32). Similar to previous findings, the researchers revealed that rTMS over bilateral DLPFCs enhanced action naming in these two groups. Moreover, they noted significantly improved object naming accuracy in participants with moderate-to-severe AD but not in participants with mild AD. However, these two studies have adopted only single rTMS sessions to evaluate the immediate cognitive effects on patients with AD. The long-term cognitive effect on patients with AD remains unknown. Therefore, they further conducted a multiple-baseline trial of 10 patients with AD divided into two groups (33). The first group underwent high-frequency (20 Hz) rTMS over the left DLPFC, five times a week for 4 weeks, whereas the second group received placebo rTMS for 2 weeks, followed by high-frequency rTMS for 2 weeks. After 2-week therapy, the authors observed participants receiving real rTMS had significant higher rates of correct responses than those receiving placebo rTMS. In addition, the researchers noted that 8 weeks after the end of treatment, both groups still had lasting improved performance (Table 1).

**Table 1:** Clinical trials using rTMS as therapeutic tool in Alzheimer's disease.

**Study (first author, year)**	**Study design**	***N***	**Primary cognition measure**	**Mean age (y)**	**Mean MMSE**	**Stimulation parameters**	**Brain target**	**Sham method**	**Main results**
Cotelli et al. (31)	Controlled study	15	Picture naming	76.6 ± 6.0	17.8 ± 3.7	20 Hz, 90% MT, 600 ms	L/R DLPFC	Coil perpendicular to the scalp	Improve action naming
Cotelli et al. (32)	Controlled study	24	Picture naming	Moderate to severe/Mild: 77.6/75.0	Moderate to severe/Mild: 14.3/19.7	20 Hz, 90% MT, 500 ms	L/R DLPFC	Coil perpendicular to the scalp	Improve action naming in mild AD and moderate-to-severe AD, but not object naming in mild AD
Cotelli et al. (33)	Double-blinded, cross-over, controlled trial	10	Auditory sentence comprehension^a^	Real-real/Placebo-real: 71.2/74.4	Real-real/Placebo-real: 16.2/16.0	20 Hz, 100% MT, 2000 stim/s for 2 weeks	L DLPFC	N/R	Improve performance with respect to baseline or placebo
Bentwich et al. (34)	Open label study	8	ADAS-cog, CGIC	74.5 ± 4.4	22.9 ± 1.7	rTMS+cognitive training(COG), 90% RMT, 10 Hz, 20 pulses x 20 trains, 2 sessions/weeks for 3 months	Six brain regions^b^	N/R	Improve ADAS-cog and CGIC after 6 weeks and 4.5 months, compared with baseline
Ahmed et al. (36)	Double-blinded, cross-over, controlled trial	45	MMSE	68.4	14.84 ± 5.5	Group 1: 20 Hz, 90% MT, 5 s, 20 trains, ITI = 25 s for 5 days; Group 2: 1 Hz, 100% MT, 2000 pulses in 2 trains, ITI = 30 s for 5 days	Bilateral DLPFC	Group 3: Coil angled away from the head	High-frequency (20 Hz) rTMS improved significantly than low-frequency (1 Hz) and sham
Rabey et al. (37)	Double-blinded, cross-over, controlled trial	15	ADAS-cog	Stim/Placebo: 72.6/75.4	Stim/Placebo:22/22	90% MT for Broca's area. L/R DLPFC 110% MT for Wernicke's area, L/R PSAC Two brain regions: 20 trains, consisting of 2 s of 10 Hz (20 pulses/train), third region: 25 trains, consisting of 2 s of 10 Hz (20 pulses/train), totaling 1,300 pulses.	Broca's area, L/R DLPFC, Wernicke's area, L/R PSAC	Sham coil	1-hour dayly rTMS-COG significantly improved ADAS-cog and CGIC than sham
Eliasova et al. (39)	Randomized, crossover, placebo-controlled study	10	Trail making test	72 ± 8	23 ± 3.56	10 Hz, 90% MT, 50 pulses x45 trains ITI = 25 s	R IFG	Vertex stimulation	High frequency rTMS significantly improved attention and psychomotor speed
Zhao et al. (38)	Randomized, double-blind, placebo-controlled trial	30	ADAS-cog, MMSE, WHO-UCLA AVLT score	70.8 ± 5.6	22.5 ± 2.7	20 Hz, MT unknown, 1 session/day and 5 days/week for total of 30 sessions	Parietal P3/P4 and posterior temporal T5/T6 according to EEG 10-20 system	Sham coil	Significantly improved ADAS-cog, MMSE and WHO-UCLA AVLT score compared with baselines, and at 6 weeks after treatment

*ADAS-cog, Alzheimer Disease Assessment Scale-cognitive subsection; DLPFC, dorsolateral prefrontal cortex; IFG, inferior frontal gyrus; ITI, inter-train interval; L, left; MMSE, Mini-Mental State Examination; N/R, not reported; PSAC, parietal somatosensory association cortex; R, right; Ref, reference electrode; MT, motor threshold; rTMS, repetitive transcranial magnetic stimulation; sham, sham group; stim, stimulation group; TC, temporal cortex. WHO-UCLA AVLT, World Health Organization University of California-Los Angeles, Auditory Verbal Learning Test*.

a *The auditory sentence comprehension subtest from the Battery for Analysis of Aphasic Deficits*.

b *Six brain regions: Broca's area, Wernicke's area, the right DLPFC, left DLPFC, and right and left parietal somatosensory association cortices (R-pSAC and L-pSAC, respectively)*.

In Egypt, Ahmed and colleagues recruited 45 participants with AD and randomly assigned them into three groups (36). The first group underwent five sessions of high-frequency (20 Hz) rTMS over the DLPFC. The second group received low-frequency (1 Hz) rTMS, while the third group underwent sham rTMS. The results showed a significant improvement in MMSE after applying high-frequency (20 Hz) rTMS. In addition to the DLPFC, Eliasova and colleagues applied high-frequency (10 Hz) rTMS over the right inferior frontal gyrus (IFG) (39). Ten participants with early AD underwent 10 Hz rTMS over the right IFG and vertex in random order, 2250 stimuli per session. The authors found a significant improvement in in the Trail Making Test parts A and B after applying 10 Hz rTMS over the right IFG.

In China, Zhao and colleagues included 30 participants with AD, and assigned 17 participants into the rTMS group, 13 participants into the sham group (38). Patients of the rTMS group underwent 30 sessions of 20 Hz rTMS over three brain regions for 6 weeks, whereas the control group received placebo stimulation. Three brain regions included posterior temporal T5/T6 and parietal P3/P4, based on the 10–20 electroencephalogram system. The authors found a significant improvement in ADAS-Cog, MMSE, as well as World Health Organization and University of California–Los Angeles Auditory Verbal Learning Test after applying rTMS over the three brain regions for 6 weeks.

In Israel, Bentwich and colleagues developed a combined treatment of high-frequency rTMS and cognitive training (rTMS-COG) (34). Eight patients with AD underwent daily rTMS-COG treatment for 6 weeks, and then maintained two sessions per week for 3 months. High-frequency rTMS were delivered over six specific brain regions including Broca's area, Wernicke's area, bilateral DLPFC, and right and left parietal somatosensory association cortices (R-pSAC and L-pSAC, respectively). The average ADAS-Cog scores significantly improved from 22.5 at baseline to 18.3 at 6-week and 18.5 at 4.5-month. Clinical Global Impression of Change (CGIC) scores also improved by 1.0 and 1.6 points, respectively. Based on these positive results, the same group recruited 15 patients with AD and randomly assigned them into two groups (37). Seven participants underwent rTMS-COG one hour per day, five times a week for 6 weeks, followed by two times a week for three months. Eight participants in the placebo group received sham treatment. The authors found an improvement in ADAS-Cog and CGIC after applying rTMS-COG for 6 weeks and 4.5 months. However, the effects of rTMS and cognitive training are difficult to differentiate because these trials lacked a control group receiving only cognitive training. Moreover, they stimulated six brain regions instead of only the DLPFC. The cognitive effects of stimulation on different regions remain unclear. Therefore, a recent review suggested that further trials are required to use a larger sample size to investigate the synergic effects of rTMS and cognitive training and investigate the cognitive effects on different brain regions (42).

### Transcranial direct current stimulation

The transcranial direct current stimulation (tDCS) delivers electric current, typically ranged 1 to 2 mA, through two or more electrodes placed on the scalp (43). Researchers put anodal and cathodal electrodes into holding bags and moisten them with saline or conductive gel to lower electric resistance. Two electrodes are placed over the head based on the international 10–20 point system. This week current penetrates skull and modulates neural activity of targeted brain regions (44). Generally, anodal tDCS increases cortical excitability in the brain region under and around the placement, while cathode tDCS decreases (45). tDCS may modulate neuronal activity with polarity change to altering membrane polarization (46, 47). Besides, tDCS is safe (48), tolerable, and low cost for patients, therefore, the studies with tDCS use have grown in decades.

A number of small trials have suggested that tDCS may enhance specific cognitive functions in patients with AD (49–55). Ferruci and colleagues enrolled 10 patients with probable AD, who received anodal, cathodal or sham tDCS in three sessions (49). These patients underwent stimulation over the bilateral temporoparietal (TP) with a current intensity of 1.5 mA for 15 min/session. When patients underwent sham tDCS, they received stimulation for only 10 s. The authors found anodal tDCS improved word recognition task accuracy after stimulation compared with baseline (17.9 vs. 15.5,*p* = 0.0068). But cathodal tDCS significantly worsened it, while sham tDCS left it unchanged from baseline (Table 2).

**Table 2:** Clinical trials using tDCS as a therapeutic tool in Alzheimer's disease.

**Study (first author, year)**	**Study design**	***N***	**Primary cognition measure**	**Mean age (y)**	**Mean MMSE**	**Stimulation parameters**	**Brain target**	**Sham method**	**Main results**
Ferrucci et al. (49)	Crossover design	10	Word recognition	75.2 ± 7.3	22.7 ± 1.8	anodal tDCS, 1.5 mA, 15 min	Bilateral temporopariatel areas, Ref: R deltoid	Stimulation was delivered for 10 s	Anodal tDCS improved accuracy of the word recognition memory task
Boggio et al. (50)	Crossover design	10	VRM	79.1 ± 8.8	17.0 ± 4.9	anodal tDCS, 2 mA, 30 min	(1) L DLPFC, (2) L TC, Ref: R supraorbital area	Stimulation was delivered for 30 s	Temporal and prefrontal tDCS improved VRM as compared with sham stimulation.
Boggio et al. (51)	Crossover design	15	VRM	71.1 ± 5.8	Anodal/Sham: 20.3/19.2	anodal tDCS, 2 mA, 30 min for 5 days	Bilateral temporal regions, Ref: R deltoid	Stimulation was delivered for 30 s	Temporal anodal tDCS for 5 days improved VRM and the improvement persists for at least 4 weeks after therapy.
Cotelli et al. (55)	Randomized, double-blind placebo-controlled	24	Face-name association task	76.6/74.7/78.2^a^	20.1/20.8/22.1^a^	anodal tDCS 2 mA, 25 min/day, 5 days/week for 2 weeks	L DLPFC, Ref: R deltoid	Stimulation was delivered for 40 s	Both Group 1 (the anodal tDCS plus individualized computerized memory training) and Group 2 (the placebo tDCS plus individualized computerized memory training) significantly improved performances at 2 weeks compared with Group 3 (the anodal tDCS plus motor training).
Khedr et al. (52)	Randomized, double-blind placebo-controlled	34	MMSE	69.7 ± 4.8	18.1 ± 3.3	(1) anodal tDCS, (2) cathodal tDCS 2 mA, 25 min/day for 10 days	L DLPFC, Ref: R supraorbital area	Stimulation was delivered for 30 s	Both anodal and cathodal tDCS improved MMSE scores compared with sham tDCS
Bystad et al. (53)	Randomized, double-blind placebo-controlled	25	CVLT-II	Active/Placebo:70.0/75.0	20.0/21.2	anodal tDCS 2 mA, six 30-min sessions for 10 days	Left temporal cortex	Stimulation was delivered for 30 s	No significant difference between the active and placebo groups in neurocognitive tests

*ADAS-cog, Alzheimer Disease Assessment Scale-cognitive subsection; atDCS, anodal transcranial direct current stimulation; ctDCS, cathodal transcranial direct current stimulation; DLPFC, dorsolateral prefrontal cortex; L, left; MMSE, Mini-Mental State Examination; min, minute; N/R, not reported; R, right; Ref, reference electrode; s, second; sham, sham group; stim, stimulation group; TC, temporal cortex; tDCS, transcranial direct current stimulation; CVLT-II California Verbal Learning Test–Second Edition; VRM, Visual recognition memory*.

a *atDCS plus memory training group/ placebo tDCS plus memory training group/ atDCS plus motor training group*.

Boggio and colleagues demonstrated that anodal tDCS over the left DLPFC and temporal cortex improved Visual Recognition Memory (VRM) in patients with AD (50). The researchers enrolled 10 AD participants who received three sessions including two real stimulations and one sham stimulation. These patients underwent real stimulation over the left DLPFC or temporal cortex with a current intensity of 2 mA for 30 min per session. The sham stimulation was applied only for the first 30 s. The authors assessed the neuropsychological tests during tDCS stimulation. They found tDCS over left DLPFC and temporal cortex significantly improved VRM tasks. The same group revised their study design, which allowed them to evaluate the long-lasting outcome of tDCS (51). They applied tDCS bilaterally over the temporal regions through two scalp anodal electrodes. These patients received stimulation over the bilateral temporal regions with a current intensity of 2 mA for 30 min a day, 5 days a week. After 5-day treatment, a significant improvement in VRM was observed, and the improvement maintained for 1 month after treatment. However, no significant improvement in visual attention or general cognitive performance was found.

In Egypt, Khedr and colleagues included 34 participants with AD and randomly assigned them to three groups. Participants of the anodal group and the cathodal group underwent daily stimulation for 10 consecutive days, with a current intensity of 2 mA for 25 min/day. The authors observed a significant improvement on MMSE scores after applying anodal or cathodal tDCS for 10 days (52).

In addition to tDCS, Cotelli and colleagues developed a combined therapy of anodal tDCS with individualized computerized memory training or motor training (55). The authors recruited 36 participants with AD and randomly assigned them to three groups. Patients in the first group underwent anode tDCS and individualized computerized memory training, while those in the second group received placebo tDCS and individualized computerized memory training. Participants in the third group underwent anodal tDCS and motor training. The tDCS stimulation was applied over the left DLPFC with a current intensity of 2 mA for 25 min a day, 5 days a week, for 2 weeks. Their findings showed a significant improvement on Face-Name associations in AD patients receiving individualized computerized memory training.

However, the results of Bystad and colleagues are inconsistent with previous findings. The authors recruited 25 AD patients and randomly assigned them into active tDCS group or placebo tDCS group. Patients underwent six sessions of stimulation over the left temporal cortex for 10 days, with a current intensity of 2 mA for 30 min/session. No significant difference was observed between the active tDCS group and placebo tDCS group in neurocognitive tests (53). To address these conflicting results, further trials are required to investigate different trial design, stimulation protocol, and standardized neuropsychological memory assessment.

### Transcranial alternating current stimulation

Transcranial alternating current stimulation (tACS) delivers a current which oscillates above and below zero with a given stimulation strength (i.e., peak-to-peak amplitude) at a particular frequency (56). In tDCS, the excitability thresholds of neuronal membrane potentials are modulated (44, 57), whereas tACS directly interacts with ongoing neuronal activity during cognitive or sensory-motor processes, leading to an entrainment or synchronization of brain network oscillations (56–59).

In previous studies, stimulation frequencies have been chosen within the range of the human electroencephalography frequency band and close to the frequency of the predominant oscillations of neuronal networks and cognitive processes (56, 60, 61). Brain oscillations represent various brain functions. Because specific frequencies reflect particular ongoing cognitive or sensory-motor processes (56, 60), tACS may enhance ongoing processes through exogenous augmentation of those oscillations (60, 62). Therefore, tACS has the potential to synchronize frequency-specific neuronal networks, thereby causing behavioral changes (61). Moreover, tACS may have the potential to infer causal associations between brain oscillations and cognitive processes (57, 60, 61).

Small trials have shown that tACS can improve specific cognitive functions in healthy adults by directly interacting with ongoing oscillatory cortical activity (56, 60, 61). For example, a sham-controlled crossover trial of 24 healthy adults reveled that tACS significantly improved retrieval accuracy (63). Therefore, tACS may also have potential effects on patients with AD. However, no study with tACS in AD has been published in PubMED. One trial of tACS for AD patients is registered at clinicaltrials.gov. Further trials are required to investigate the potential roles of tACS for cognitive enhancement in patients with AD.

### Electroconvulsive treatment

Cerletti et al. first used electrical stimulation to cause convulsions in a patient with schizophrenia who was experiencing delusions and hallucinations (64). They reported that they restored the patient to “clear-headedness” and health. Ever since, numerous electroconvulsive treatment (ECT) trials have been conducted for psychiatric disorders. ECT has been approved by the US FDA to treat major depression, mania, schizophrenia, and catatonia (65). Whether ECT-induced seizures can result in cognitive impairment is debated. Most adverse cognitive effects of ECT last a short amount of time. Modifications of and improvements to treatment techniques have been implemented to minimize cognitive side effects (66).

Numerous studies and many meta-analyses have shown gray matter atrophy and lower levels of brain-derived neurotrophic factor (BDNF) are associated with AD (1, 2). Moreover, a meta-analysis revealed that ECT may increase BDNF levels in depressed patients (67). Studies have investigated APOE-ε4 and beta amyloid level after ECT treatment, but the findings are contradictory (68). Moreover, in a trial of ECT for depression, gray matter, and hippocampus volume were reported to increase following ECT (69).

Kumar et al. reviewed 5,154 publications and suggested ECT may improve long-term cognitive outcomes in late-life depression (LLD) (70). For example, Hausner et al. included 44 elderly inpatients with MDD, and divided these patients into three groups (dementia group: 12 subjects, MCI group: 19 subjects, no cognitive impairment (NCI) group: 13 subjects) (71). They delivered right unilaterally at minimal 250% seizure threshold or bilaterally at minimal 150% seizure threshold, two to three times per week. In the dementia group, the pre-ECT MMSE = 22.7 (4.4) and the post-6 month MMSE is 25.6 (3.0). Verwijk et al. included 42 depressed patients aged ≥55 years (72). They found improvement in the Trail Making Test-A (76.21 vs. 61.63, *p* = 0.024) and Letter Fluency Test (9.00 vs. 12.50, *p* = 0.004) but not in the MMSE after 6 months. Besides, a retrospective cohort study of 126 patients with ECT treatment reported that the MMSE score was significantly higher at the 6-month compared with baseline (27.96 vs. 26.25, *p* < 0.01) (73). However, these studies addressed elderly depression instead of AD. One trial of ECT for AD patients has been registered at clinicaltrials.gov. Further studies of ECT in patients with AD are required.

### Magnetic seizure therapy

Magnetic seizure therapy (MST) is a new implementation of TMS. It is based on the rationale of ECT. Similar to ECT, MST induces seizures using high-intensity rTMS, but with greater control. One study demonstrated the antidepressant effect of MST and identified a response rate of approximately 50–60% (74). A study of 10 patients with refractory depression reported that the relative glucose metabolism increased in brain regions including the medial frontal cortex, orbitofrontal cortex, basal ganglia, and DLPFC after MST treatment (75). This indicated that the mechanism of MST treatment may be associated with these activities in these brain regions.

A systematic review of 11 MST trials revealed little to no adverse cognitive effects (76). Moreover, Kosel et al. identified significantly-improved verbal learning performance in refractory depressed patients (77). Besides, Lisanby et al. found that MST improved the velocity and accuracy of visual cancellation tasks in patients with major depression (78). These studies have revealed that TMS may improve cognitive function in depressed patients. However, until now no trial with MST in AD has been published in PubMED or registered at clinicaltrials.gov. Luber et al. reviewed the applications of TMS and MST in neuropsychiatric illnesses related to cerebral aging (79). The authors suggested MST may enhance cognition or reduce amnesia. Therefore, MST warrants further exploration for its potential effect on patients with AD.

### Cranial electrotherapy stimulation

Cranial Electrotherapy Stimulation (also referred to as cranial electrostimulation [CES]) applies pulsed, low-amplitude, electrical currents (typically <1 mA) to the brain through ear clip electrodes. The US FDA has approved CES for the treating depression, anxiety, and insomnia (80).

Small trials have demonstrated that transcutaneous electrical nerve stimulation (TENS) may enhance specific cognitive function in patients with AD (81, 82). Scherder and colleagues (81) recruited 16 participants with early-stage AD, and assigned them equally into the experimental group or the placebo group. The researchers fixed two electrodes on the participant's back between Th 1 and Th 5. These patients underwent stimulation with asymmetric biphasic square impulses in bursts of trains, 30 min per day, for 6 weeks. Each trains contained nine pulses with an internal frequency of 160 Hz. The repletion rate was 2 Hz and pulse width was 40 μs. After a 6-week treatment, a significant improvement was observed in Face Recognition, Picture Recognition and Recognition subtest of the 8 Words Test.

Based on these promising findings, the same research team from the Netherlands used the same protocol in the mid-stage of AD (82). They enrolled 16 patients with mid-stage AD. The subjects of the experimental group received 30-min stimulation daily for 6 weeks. The protocol was similar to previous study. Compared with TENS in an early stage, they observed TENS caused less beneficial effects in patients in the mid-stages of AD.

Scherder and colleagues further investigated the cognitive effects of CES in AD patients, because CES mimics TENS but mediated stimulation via the patients' earlobes (head) instead of patients' back. This research team selected 18 participants with AD, and randomly assigned them into the intervention group and the control group (83). Participants of the intervention group underwent low-frequency (0.5 Hz) stimulation with an intensity of 10 to 600 μA, 30 min per day, 5 days per week for 6 weeks. However, after 6-week CES treatment, no improvement in cognition was found.

Therefore, Scherder and colleagues launched a study of high-frequency (100 Hz) CES in 21 patients with AD, and assigned theses participants into the experimental group or the control group (84). The protocol was similar to previous trial except the frequency. Patients in the intervention group underwent high-frequency (100 Hz) stimulation with an intensity of 10–600 μA, 30 min/day, and 5 days/week. However, the results revealed no cognitive improvement after 6-week treatment. Further research with large sample sizes and better designs may be required to evaluate the effect of CES on cognition.

### Non-invasive vagus nerve stimulation

Non-invasive VNS (nVNS) does not require a surgical procedure to implant an electrode. nVNS devices are portable and can stimulate the vagus nerve indirectly through the skin of neck or ear (85–87). nVNS is less expensive, carries a lower risk, and is more convenient than iVNS.

Two small trials have shown iVNS may enhance cognitive function in patients with AD (21, 22). In Sweden, researchers enrolled 10 patients with AD (22). Each patient received surgery to implant a pulse generator, which deliver programmable signals to the left-side tenth cranial nerve. The initial settings were frequency 20 Hz, pulse width 500 μs, and current 0.25 mA. The stimulation was on for 30 s followed by a pause for 5 min. After 3 months of treatment, they found the response rates were 70% (seven of 10 patients) on the ADAS-Cog and 90% (nine of 10 patients) on the MMSE. Moreover, 6 months after the iVNS treatment, response rate was still 70% on the ADAS-Cog, and 70% on the MMSE. Adverse effects of invasive VNS were transient and mild.

Therefore, based on these promising findings, the same research team recruited another seven patients with AD. The researchers followed up these total 17 patients for at least 1 year (21). They found that 1 year after iVNS treatment, the score of the ADAS-Cog in seven patients did not decline or even increase, while the score of the MMSE in 12 patients did not decline or even improve compared with baseline. These two small trials revealed that invasive VNS was well-tolerated and improved specific cognitive functions in MMSE and ADAS-Cog after 1 year and 3 months of treatment, respectively.

Non-invasive VNS may affect cognition through the same neural pathway. Until now, no trial with non-invasive NVS in AD has been published in PubMED or registered at clinicaltrials.gov. Further studies of non-invasive VNS in patients with AD are required.

## Brain targets and mechanisms in invasive brain stimulation

### Nucleus basalis of meynert

Nucleus basalis of Meynert (NBM) is the first target for DBS in AD (11). The NBM is a group of cholinergic nucleus in the forebrain (88). Previous studies have shown loss of central cholinergic neurons of the basal forebrain cholinergic system in AD patients (89–91). Moreover, in early-stage AD, volume reductions developed in posterior parts of NBM (92). The atrophy of cholinergic neurons is considered to result in cognitive impairment in AD (93). Therefore, current DBS trials are based on the hypothesis that stimulating NBM may enhance the cholinergic system and thereafter improve the cognitive functions in patients with AD (94).

### Fornix

The fornix is an integral white matter bundle in the medial diencephalon. It connects the medial temporal lobes to the hypothalamus, and serves as a vital role in the memory circuit of Papez (95, 96). Previous studies have shown that fornix lesions cause severe memory impairments (97, 98). Besides, the memory impairment and progression in AD are correlated with axonal degeneration and dysfunction in the fornix (99). Therefore, several trials were performed to evaluate the hypothesis that fornix DBS could enhance the circuit of Papez and thereafter improve memory and cognitive functions (10, 16).

### Vagus nerve

The possible mechanism for cognitive improvement through iVNS is based on the neural anatomy. The vagus nerve (tenth cranial nerve) projects to the locus coeruleus (LC), which is the major nucleus of origin for noradrenergic projections in the brain (100). Studies have revealed atrophy of the LC in patients with AD (101). Moreover, decreased norepinephrine (NE) concentration in the temporal cortex is correlated with cognitive impairment in patients with AD (102). In addition, NE can inhibit the inflammatory activation of microglial cells and functions as an endogenous anti-inflammatory agent (103). Therefore, iVNS may increase the NE concentration and decrease inflammation. These mechanisms may involve in specific cognitive functions in AD. Further trials with large sample are required to investigate this hypothesis.

## Brain targets and mechanisms in non-invasive brain stimulation

### Dorsolateral prefrontal cortex (DLFPC)

In contrast to IBS, no consensus has been made for NIBS regarding which brain region should be targeted in AD. Most NIBS studies (31–33, 36, 52, 55) including TMS, tDCS targeted the DLPFC, a region involving in the decline of working memory and specific executive functions in early AD (104, 105). Moreover, the DLPFC may enable compensatory mechanism for working memory performance, and change dynamic neuroplasticity after prefrontal cortex damage (106–108). Thus, these findings may support the use of DLPFC as a potential stimulation target to improve specific cognitive functions in patients with AD.

### Broca's area, wernicke's area, and parietal somatosensory association cortex (PSAC)

In addition to DLPFC, other cortex areas have been investigated. Broca's area, located in the left frontal part of the temporal lobe, involves sentence comprehension in articulatory rehearsal (109) whereas Wernicke's area, located in the left frontal and left posterior part of the temporal lobe, processes lexical meanings of words (110). Right parietal somatosensory association cortex (PSAC) is in the parietal lobe and associated with visual and spatial attention impairment in AD (111, 112). Two trials targeted six brain regions including right DLPFC, left DLPFC, Borca's area, Wernicke's area, PSAC, and left PSAC (34, 37). These two trials of rTMS over DLPFC, Broca's area, Wernicke's area, and PSAC reported improved ADAS-cog in patients with AD.

### Inferior frontal gyrus

Right inferior frontal gyrus (IFG) plays an important role in the right-lateralized ventral attention network governing reflexive reorienting (113, 114). Previous neuroimaging study has demonstrated that right IFG involves in dissociating inhibition, attention, and response control in the frontoparietal network (115). The lateral prefrontal cortex, particularly the right IFG, can be activated during response inhibition in the go/no-go task (116). Chambers and colleagues reported that rTMS over the right IFG could modulate stop-signal reaction time (117). Elisaova and his colleagues targeted IFG and found that rTMS may improve attention in patients with early AD (39).

### Temporal cortex

Increasing evidences have shown the association between dysfunction or atrophy of temporal cortex and Alzheimer's diseases (118). Mesial temporal lobe dysfunction is correlated with memory deficits such as episodic memory impairment (119). Boggio and colleagues reported that anodal tDCS over bilateral temporal cortex improved visual recognition memory in patients with AD (51). However, Bystad and colleagues found no significant improvement after tDCS over left temporal cortex (53). The inconsistence may be caused by anatomical differences, limited sample size, severity of AD, and different neuropsychological tests (53). Further studies are suggested to evaluate these differences.

### Temporopariatel cortex

In addition to temporal cortex, hypofunction or atrophy of temporopariatel (TP) cortex has been noted in AD (120, 121). Both pilot trials of rTMS and tDCS over TP areas have shown promising results. Zhao and colleagues applied rTMS over TP cortex and found a significant improvement in cognitive and language function (38). Ferrucci and colleagues reported that tDCS over TP areas can improve recognition memory performance in patients with AD (49).

In summary, current invasive DBS studies aimed at the subcortical areas such as NBM and fornix, while non-invasive DBS studies aimed the cortical areas such as DLFPC, temporal cortex, Broca's are, Wernicke's area, and PSAC. Generally speaking, the subcortical area is associated with emotion and behavior (122), whereas cortical function is related to cognition (123). However, whether the stimulated regions meet the outcome variables are not clearly evaluated and understood. Further studies are needed to evaluate the targeted brain areas and cognitive outcomes.

### Stimulation therapy combined with cognitive training or cognitive-challenging activities

Two trials of rTMS combined with cognitive training (rTMS-COG) have shown promising results in patients with AD (34, 37) and suggest synergistic effects better than rTMS therapy or cognitive training alone. However, the synergistic effects of rTMS-COG are unclear due to the lack of a control group with cognitive therapy only. Therefore, this made it difficult to differentiate the effects between rTMS and cognitive therapy. Furthermore, they applied rTMS over six brain areas including DLPFC. This also made it difficult to compare with other rTMS trials over one or two brain areas. Further controlled-design, larger, multi-center studies are needed.

### Practical and ethical challenges

IBS treatments, especially DBS, need surgical procedures and cause more concerns about the safety and ethical issues. A few pilot studies have reported that the surgery was well-tolerated with no adverse effects (10, 16, 124), but an AD trial with DBS over fornix has noted four acute serious safety events and three long-term serious events (15). Because DBS surgery and stimulation may cause neurologic and psychiatric side effects, and patients with AD tend to have more comorbidities than normal aged population, several reviews have raised the ethical considerations about participants selection, decision-making procedure, and informed consent (124, 125).

On the other hand, NIBS therapies such as rTMS and tDCS led to less safety and ethical concerns. rTMS may cause mild headache, tinnitus, short-term hearing loss, short-term memory change, or acute psychiatric effects. All these adverse effects are transient and disappear after turning off (25). The most serious side effect is seizure, but the incidence is rare. In a study that reviewed trials by rTMS over non-motor areas between 1998 and 2003, only two seizure cases were found in total 3,092 participants (126). Similarly, tDCS may cause relatively minor adverse effects including fatigue, mild headache, nausea, or itching (127, 128).

### Improving cognition indirectly by improving depression

Previous studies of ECT and MST in depressed patients have shown improvement not only in depressive symptoms but also in cognitive functions (71, 72, 77, 78). However, those trials investigated older participants with depression instead of participants with AD. Therefore, the improvement in cognition may correlate with the improvement in depressive symptoms. Whether ECT or MST can improve cognition directly or indirectly remains unclear. Further studies are needed to explore the underlying indirect mechanisms in AD.

## Conclusions

Studies are increasingly investigating brain stimulation techniques as novel therapeutic approaches to AD. Although some studies have revealed promising results, many lack large samples and the appropriate power, or are poorly designed and not hypothesis-driven. This review examined IBS therapies, namely DBS and invasive VNS, and NIBS therapies, namely TMS, tDCS, tACS, ECT, MST, CES, and non-invasive VNS. Because many brain stimulation methods have no standard settings and guidelines, a robust comparison of these trials remains incomplete. However, stimulation-associated improvements in memory and specific cognitive functions are promising. Moreover, stimulation that is targeted at multiple brain regions or combined with other treatments such as cognitive training appear to produce more positive effects. Therefore, although the field of brain stimulation is relatively immature, such techniques, especially rTMS, warrant further study for their therapeutic implications on patients with AD.

## Author contributions

C-HC drafted the initial manuscript. H-YL provided expert opinions and reviewed the final submitted manuscript. C-HL critically reviewed the draft of manuscript, and approved the final submitted version manuscript.

### Conflict of interest statement

The authors declare that the research was conducted in the absence of any commercial or financial relationships that could be construed as a potential conflict of interest.
